# Histopathology of Corneal Lenticules Obtained from Small Incision Lenticule Extraction (SMILE) versus Microkeratome Excision

**DOI:** 10.18502/jovr.v18i1.12722

**Published:** 2023-02-21

**Authors:** Salwa Abdelkawi Ahmed, Ibrahim Mohi Eldin Taher, Dina Fouad Ghoneim, Mohammed Ahmed Elnaggar, Aziza Ahmed Hassan

**Affiliations:** ^1^Department of Vision Science, Biophysics and Laser Science Unit, Research Institute of Ophthalmology, Giza, Egypt; ^2^Ophthalmic Unit, National Institute of Laser Enhanced Science, Cairo University, Egypt

**Keywords:** Comet Assay, DNA Fragmentation, Femto SMILE, Fourier Transform Infrared Spectroscopy, Histological Analysis

## Abstract

**Purpose:**

To study the alterations on the lenticules extracted after femtosecond (Femto) small incision lenticule extraction (SMILE) versus the corneal free cap removed using a microkeratome.

**Methods:**

The visuMax (500 kHz; laser energy: 180 nJ) was used for small-incision lenticule extraction. Free caps from human cadaveric corneas were excised by microkeratome. The collected lenticules were examined with the light and transmission electron microscope (TEM) for histological analysis, DNA fragmentation was assessed by agarose gel electrophoresis, DNA damage was evaluated using comet assay, and corneal proteins secondary structure was assessed by Fourier transform infrared spectroscopy (FTIR).

**Results:**

Light microscopic examination showed the presence of more edematous stroma under Femto SMILE than under free cap with a percentage change of 101.6%. In the Femto SMILE group, TEM examination showed pyknotic keratocytes, disruption, and cavitation of the collagen arrays stromal area under Femto SMILE. The DNA fragmentation for the Femto SMILE group revealed one undefined band with a size of 1.1 Kbp. The comet assay analysis indicated the presence of 3% and 8.0% tailed cells for the free cap and Femto SMILE groups, respectively. The tail lengths were 1.33 ± 0.16 and 1.67 ± 0.13 µm (*P* < 0.01), the percentage of tail DNA was 1.41 ± 0.18% (*P* < 0.01) and 1.72 ± 0.15%, and the tail moments were 1.88 ± 0.12 AU and 2.87 ± 0.14 AU (*P* < 0.001) for the free cap and Femto SMILE groups, respectively. FTIR spectroscopy of the Femto smile group revealed disorders in the secondary and tertiary structure of the proteins.

**Conclusion:**

Femto SMILE technique induced more structural changes, DNA fragmentation, DNA damage, and corneal proteins secondary structure alteration than those induced by a microkeratome cutting. These changes may be attributed to the deep penetration of high energy levels to the corneal layer. These findings may highlight the potential impact of the Femto SMILE on the cornea and the necessity for managing the laser parameters used.

##  INTRODUCTION

Recently, considerable attention has been paid to the Femtosecond laser (FS) in the infrared range (1053 nm and pulse duration 10 ${}^{--15}$ s). FS laser causes photodisruption and photoionization effects on sensitive ocular tissues such as cornea,^[1]^which generate an intensifying cloud of ionized molecules and free electrons that disrupt the treated tissue.^[2]^ The femtosecond (Femto) small incision lenticule extraction (SMILE) technique in refractive surgery has excellent cutting accuracy and safety as compared with the microkeratome flap creation in laser in-situ keratomileusis.^[3]^

FS laser has been employed in many applications, such as laser-assisted anterior and posterior lamellar keratoplasty, excision of donor buttons in endothelial keratoplasty, customized trephination in penetrating keratoplasty, wound construction, capsulorhexis, and cataract surgery.^[4,5,6]^ In addition, FS laser is used in corneal refractive surgeries such as LASIK flap creation, implantation of intrastromal corneal ring segments, astigmatic keratotomy, femtosecond lenticule extraction (FLEx), intrastromal presbyopia correction, and small-incision lenticule extraction (SMILE).^[7,8]^

The SMILE technique has gained importance for its application in corneal refractive surgery. It is performed entirely with the FS laser, without flap creation, avoiding all flap-related complications and reducing the inflammation and postoperative dry eye syndrome.^[9,10,11]^

Previous researches on the Femto SMILE technique were focused only on the ultrastructure change or the efficacy and safety at the clinical levels without considering the change in the protein levels or the genetic damage of lenticules.^[12,13,14]^

Several studies have investigated the refractive, optical, and histological outcomes of Femto smile procedures.^[15]^ Other studies determined the best cutting parameters by using high frequency, small spot distances, and low pulse energy.^[16,17]^

The earliest scanning electron microscope study (SEM) showed smooth cuts by applying 500 nJ pulse energy, 200 kHz, and a spot distance of 5.0 μm (WaveLight UltraFlap - Femtosecond laser).^[18]^ However, using 200 kHz, 185 nJ pulse energy, and 9.0 μm^2^ spot separation (VisuMax laser) showed lower surface quality for the lenticules of an animal model than those obtained with a mechanical microkeratome.^[19]^

Although using a higher frequency at 500 kHz (VisuMax femtosecond), 130 nJ pulse energy, and 9.0 μm^2^ spot separation improved the surface quality viewed in 12 cadaveric eyes, cavitation bubbles and rough patches were still seen in their samples.^[12,20]^

In another recent study by Ziebarth et al using the VisuMax femtosecond laser at 500 kHz, 130 nJ pulse energy, and a closer spot separation of 2.5 × 2.5 μm, the procedure showed an easy extraction and smooth lenticules surfaces.^[20,21]^ Another study by Ang et al on 30 Femto SMILE lenticules of human corneas compared with the microkeratome cuts assessed by the SEM and TEM^[12,21]^ found that Femto SMILE lenticules showed more surface roughness, tissue bridges, and cavitation changes which was in contrast with the study of Ziebarth et al.^[20,21]^ A comparison among those three previous studies on SMILE lenticules is not possible, as different spot sizes were used.^[12,20][21]^

The current study aimed to evaluate the morphological differences, DNA fragmentation, DNA damage, and the deformation in proteins' structure in human corneal lenticules after the Femto SMILE laser procedure versus the corneal free cap procedure using the microkeratome.

##  METHODS

Ten corneas of human cadavers' donors were obtained from Al-Kaser Elaini eye bank. The mean donor age was 50 ± 10 years, the death-to-tissue harvest time was < 24 hr, and the mean time from death to the surgical procedure was 6 ± 5 days. Ten myopic patients (–4 D to –9 D) were assigned for Femto SMILE laser (*n* = 20 eyes) using VisuMax femtosecond laser system for SMILE at 100 μm depth. This work was conducted per the Declaration of Helsinki as revised in 2013 and accepted by the local Scientific Research Ethics Committee of our institute (Approval no. CU-NILES/01/22). The removed lenticules were examined by light microscopy, transmission electron microscope (TEM), agarose gel electrophoresis for DNA fragmentation analysis, comet assay for DNA damage, and Fourier transform infrared spectroscopy (FTIR) for analysis of corneal proteins secondary structure.

### Creation of Free Caps from the Donor Cornea

The donor cornea was placed with the epithelial side facing upward on the tissue pedestal of the artificial anterior chamber (Coronet, Network Medical product Ltd., UK). Centration of the cornea was adjusted by using the cross-hair, and the tissue retainer was selected. The tissue retainer was dropped gently onto the tissue pedestal, and the compression head was placed over the tissue retainer and secured. As soon as the desired pressure was achieved, the flow regulator was locked. Free caps with a thickness of 130 μm were created using a microkeratome (Moria Surgical M2, Antony, France) and stored in cornea storage medium “Optisol” ( Bausch & Lomb Incorporated – Rochester, NY, USA) at 4ºC.

### Small-incision Lenticule Extraction (Smile) 

Femto SMILE operations were performed using the VisuMaxⓇ femtosecond laser system (Carl Zeiss Meditec AG, Jena, Germany) with a laser frequency of 500 kHz and laser energy of 180 nJ and multiple pulses duration of 220–280 fs. The obtained cap thickness was 130 µm, 7.5 mm cap diameter, and 6.3 mm optical zone of lenticule.^[20]^

### Histological Examination

The cornea specimens were fixed by immersion in formaldehyde (4%) and glutaraldehyde (1%) for 3 hr in phosphate buffer solution (PBS, pH 7.2) at 4ºC. Then, samples were post-fixed in 2% Osmic oxide in the PBS at 4ºC for 2 hr. After washing the specimen in the PBS and dehydrating at 4ºC using a graded series of ethanol, they were embedded in pre-labeled (plastic) capsules to be polymerized for 48 hr. The specimens were trimmed and cut to semi-thin sections (1 μm) using Lab Knife Blade (LKB) ultra-microtome to be visualized by the light microscope. For TEM, thin sections were cut (30–100 nm) using an ultramicrotome and mounted on copper grids. Next, the grids were stained with uranyl acetate for 20 min and lead citrate for 10 min. The grids were then examined by JEOL 100 CX TEM (JEOL Inc., Peabody, Massachusetts, USA). The area percentage and the means ± standard deviation of the edematous areas in each group were measured using ImageJ software (NIH, USA).^[22]^

### DNA Fragmentation Analysis 

Agarose gel electrophoresis was employed to compare fragmented and intact DNA fractions of the human corneal lenticules according to Dash et al.^[23]^ Twenty microliters of the DNA samples and markers were loaded on the 1% agarose gel wells containing 0.5 mg/ml of ethidium bromide in TBE buffer (pH 8.0). A gel electrophoresis device** (**Model Horizon 58, Gibco BRL, USA) was used to run the electrophoresis and viewed using a Bio-Rad ${}^{TM}$ gel documentation system with the analysis software Quantity OneⓇ.

### Comet Assay for DNA Damage

Comet assay was employed according to Olive and Banáth to evaluate the extent of DNA damage.^[24]^ Briefly, corneas were minced in phosphate buffer saline (Ca2+- and Mg2+-free) to form a single-cell suspension (2 × 10^5^ cell/ml) and kept in an ice-cold medium. Next, agarose slides were prepared by covering the slides with a thin layer of 1% low-gelling-temperature agarose (Sigma Uldrich, USA) and allowed to dry.

The cornea cells suspension (0.4 ml) was mixed with 1.2 ml of 1% agarose at 40ºC. Then, 1.2 ml from the mixture was spread on the precoated agarose slides and allowed to solidify.

Cell lysis was performed using a cold fresh alkaline solution (pH > 13) at 4ºC overnight in the dark. The slides were removed and rinsed with an alkaline solution (pH > 13) for 20 min. Then, the slides were immersed in the electrophoresis chamber and operated for 25 min at 0.7 V/cm, 40 mA, and 20 V.

The slides were then removed and stained with propidium iodide solution (2.5 µg/ml) for 20 min (Sigma Uldrich; USA). Next, the DNA comets were visualized and photographed using a Nikon Optiphot-2 epifluorescence microscope with an attached camera (Sony CCD-IRIS, Minato, Tokyo, Japan) and connected to comet assay II software (Perceptive Instruments, UK). The software analyzes duplicate comet images for tail length (µm), percent of DNA in the tail (%), and tail moment. The results of the tail length, % tail DNA, and tail moments were calculated as the mean and standard deviation (Mean ± SD) for all experimental groups. A one‑way analysis of variance and the Student (*t*) test were employed to assess the contrast between the free cap and Femto SMILE groups.

**Figure 1:**
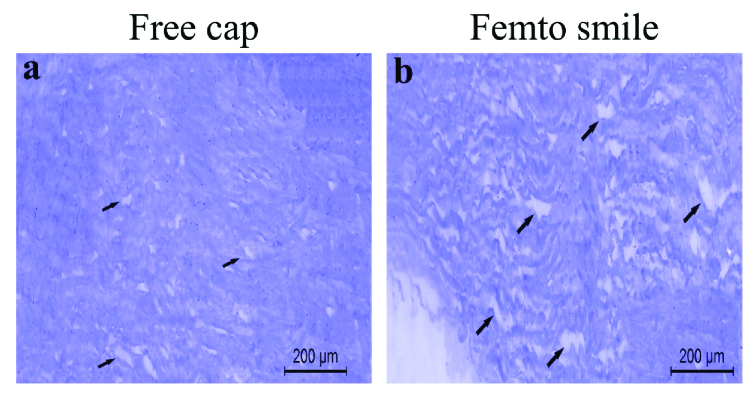
Light microscope picture for (a) free cap showing slight stromal edema and (b) Femto SMILE showing homogeneous and more edematous stroma (arrows).

**Figure 2:**
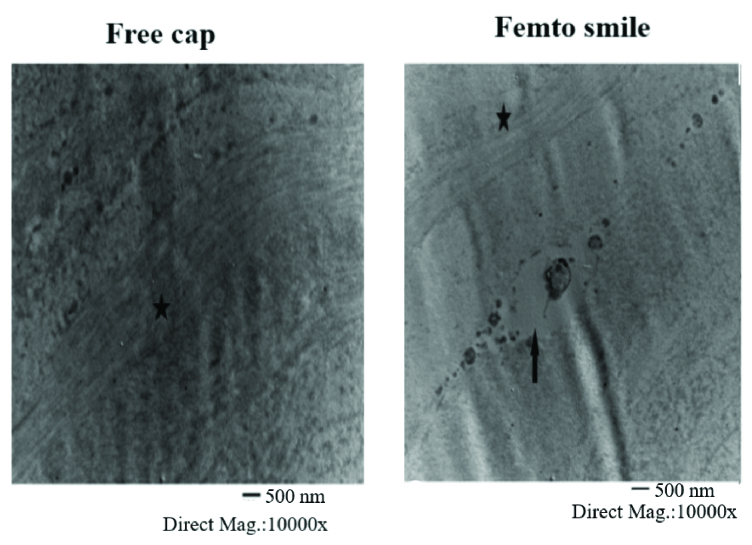
Transmission electron micrograph of (a) free cap showing more regular arrangement of the stromal lamella and intact collagen bundles and (b) Femto SMILE showing disruption of the collagen arrays (*) as well as pyknotic keratocyte (arrow).

**Figure 3:**
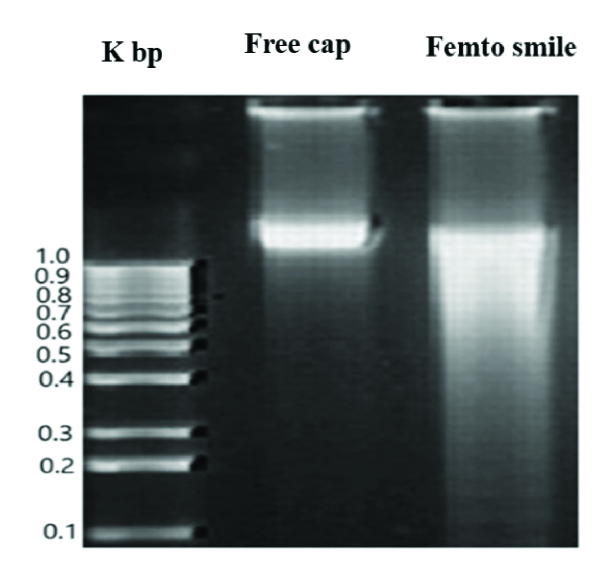
0.1% Agarose gel electrophoresis for analysis of DNA fragmentation showed a well-defined band for the free cap and an undefined band for the Femto SMILE group. The first band represents the DNA markers. Kbp, kilo base pair.

**Figure 4:**
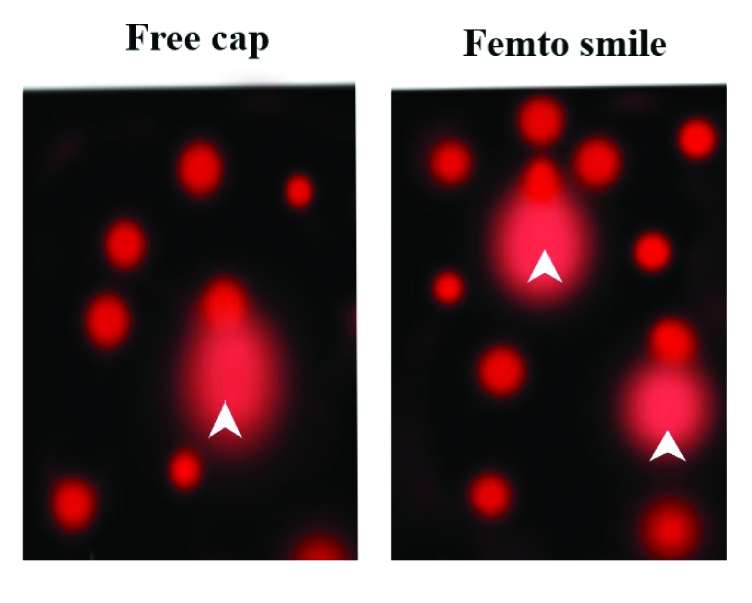
Representative Comet images of human cornea for free cap and Femto SMILE group showing a circular head corresponding to the undamaged DNA that remains in the cavity and a tail of damaged DNA (arrow head). Propidium iodide solution (2.5 µg/ml) gel Stain, Scale bars: 100 µm.

**Figure 5:**
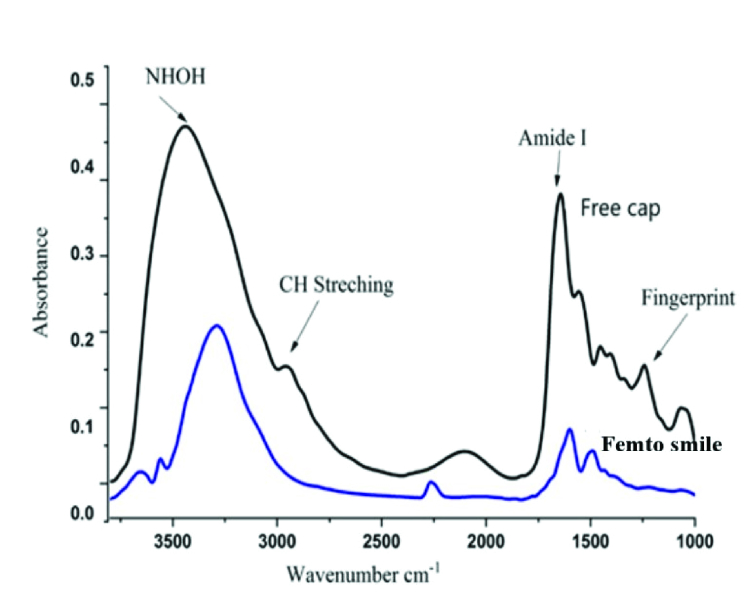
FTIR spectroscopic analysis for the corneal proteins showing the difference between the spectral profile for the free cap group and Femto SMILE groups in the NH-OH (3000–3700 cm ${}^{-1}$ ), CH stretching (2800–3000 cm ${}^{-1}$ ), Amide I (1700–1800 cm ${}^{-1}$ ), and fingerprint regions (1000–1800 cm ${}^{-1}$ ).

### FTIR Spectroscopic Analysis of Corneal Proteins

In order to verify the validity of both agarose electrophoresis and comet assay, FTIR spectroscopy for corneal proteins was carried out. FTIR spectroscopy delivers evidence about the secondary and tertiary structure of corneal proteins by aiming radiation into the infrared range which is absorbed by the sample. Each sample has a distinct group of absorption bands in its infrared spectrum. The most characteristic bands found in the infrared spectra of corneal proteins and polypeptides are Amide I and Amide II. These ascend from the amide bonds that link the amino acids. The absorption related to the Amide I band leads to stretching vibrations of the C = O bond of the amide, and absorption related to the Amide II band leads primarily to bending vibrations of the N–H bond. Both C = O and the N–H bonds are involved in the hydrogen bonding between these two different elements of the secondary structure. Moreover, the change in the vibrational frequencies of both the Amide I and Amide II bands are sensitive to the changes in the secondary and tertiary structure of the corneal proteins.^[25]^

Tertiary structure of the protein is originated when the elements of the secondary structure are arranged tightly together to form the well-defined three dimensional shape. The sidechains buried inside a folded protein are packed tightly together and must interact favorably in order to remain folded and function properly. Changes in the lenticules' protein tertiary structure were determined by comparing ratios of amide II to amide I bond intensities. This tertiary structural change indicates a transition from a folded to an unfolded state.^[26]^

First, corneas were lyophilized thoroughly by an instant insertion into liquid nitrogen (–196ºC). Next, the corneas were crushed into a powder by using a Teflon hand homogenizer for proteins extraction. Finally, the KBr disks were prepared by mixing the obtained corneal powder with potassium bromide (KBr) powder (98 mg KBr: 2 mg of the powdered cornea). The FTIR spectra in the range of 4000–1000 cm ${}^{--1}$ were obtained using a Thermo Nicolet iS5 FTIR spectrometer (USA), baseline corrected, and operated under continuous dry nitrogen gas.

### Statistical Evaluation

The area percentages of the edematous lenticules and their mean ± standard deviation were quantified using imageJ software analysis. The variations between the free cap and Femto SMILE groups were analyzed employing a one‑way analysis of variance and Student's *t*‑test for comparing between the two groups. The result was considered significant at *P* < 0.05.

##  RESULTS

### Histological Examination

Light microscopic examination for corneal free caps showed slight stromal edema [Figure 1a], while the Femto SMILE samples showed homogeneous and more edematous stroma (arrows) [Figure 1b]. The area percentage was 3.19% for the free cap group and 6.43% for the SMILE group with a percentage change of 101.6%. Moreover, the mean values ± standard deviation of the edematous areas for the free cap group was 7.9 ± 6.6 µm ${}^{2}$ and for the Femto SMILE group was 20.3 ± 13.6 µm ${}^{2}$ with a percentage change of 157% (*P* < 0.001).

The TEM examination for the corneal free caps showed a more regular arrangement of the stromal lamella, and the collagen fibers appeared as arranged threads in contrast to the Femto SMILE specimens that showed disruption and cavitation of the collagen arrays and pyknotic keratocyte [Figure 2].

### DNA Fragmentation Analysis

Agarose gel electrophoresis for corneal DNA of the free caps and the Femto SMILE laser is shown in Figure 3. The typical ladder pattern of DNA markers was separated into 10 fragments ranging from 1.0 Kbp to 0.1 Kbp. An intense ladder was noticed for the free caps with a size of 1.150 Kbp. In comparison, the ladder reflects one undefined band with a size of 1.1 Kbp for the Femto SMILE sample.

### The Comet Assay Analysis

The photograph of the comet assay [Figure 4] shows the circular head containing the high molecular weight of the undamaged DNA while the comet tail represents the migrating fragments of the damaged DNA (arrow head). The percentage of the tailed cell for the free cap group was less than the Femto SMILE group. Moreover, the mean tail lengths were 1.33 ± 0.16 µm and 1.67 ± 0.13 µm for the free cap and Femto SMILE groups, respectively. Additionally, the percentage of tail DNA was 1.41 ± 0.18% and 1.72 ± 0.15% for the free cap and Femto SMILE groups, respectively [Table 1]. The tail moment, which is the multiplication of the percent DNA in the tail by the tail length, was 1.88 ± 0.12 AU for free cap and 2.87 ± 0.14 AU for Femto SMILE (AU: Arbitrary unit). Furthermore, the percentage difference between the free cap group and the Femto SMILE group was 22.7% (*P* < 0.01) for the tail lengths, 19.8% (*P* < 0.01) for the percentage of DNA in the tail, and 41.7% (*P* < 0.001) for the tail moment, respectively.

### FTIR Spectroscopic Analysis for the Lenticules' Proteins 

The lenticular proteins were analyzed for the spectral regions at 3700–3000 cm ${}^{-1}$ , 3000–2800 cm ${}^{-1}$ , 1800–1000 cm ${}^{-1}$ , and 1700–1600 cm ${}^{-1}$ , corresponding to NH-OH stretching, CH stretching, fingerprint, and amide I regions, respectively. The profile of the free cap group [Figure 5] revealed the presence of two peaks centered at 3452 cm ${}^{-1}$ and 2962 cm ${}^{-1}$ corresponding to the NH-OH group and CH stretching group, respectively. Moreover, another five peaks were centered at 1558 cm ${}^{-1}$ , 1457 cm ${}^{-1}$ , 1240 cm ${}^{-1}$ , 1070 cm ${}^{-1}$ , and 1643 cm ${}^{-1}$ concerning amide II, CH2 bending, ${}_{assym}$ PO ${}_{2}{}_{,}{}_{}$ and ${}_{sym}$ PO ${}_{2}$ and amide I, respectively. In the Femto SMILE group, the NH–OH wavenumber had significantly changed and shifted to 3290 cm ${}^{-1}$ , and the CH stretching region had shifted to 2938 cm ${}^{-1}$ . Moreover, the fingerprint region showed several significant changes in wavenumber at 1558 cm ${}^{-1}$ (Amide II) and was shifted to 1530 cm ${}^{-1}$ ; 1070 cm ${}^{-1}$ corresponding to ${}_{sym}$ PO ${}_{2}$ was shifted to 1056 cm ${}^{-1}$ , the appearance of 1388 cm ${}^{-1}$ corresponding to COO ${}_{sym}{}_{}$ [Table 2]. In addition, ratios of amide II to amide I bond intensities were 0.95 and 0.93 for the free cap group and Femto SMILE group, respectively.

##  DISCUSSION 

In this study, we concluded that the histological analysis of human corneal lenticules could be a model of the alteration in the rest of the cornea. Using the light microscope, the Femto SMILE samples showed more stromal edema than the free cap samples with an area percentage of 6.43% and 3.19%, respectively. Moreover, pyknotic keratocytes, and disrupted

collagen were detected in the Femto SMILE group with TEM examination. Furthermore, both light microscope and TEM results for the lenticules have been promising as they have shown lower surface quality cavitation bubbles, more surface roughness, and tissue bridges than those obtained with a mechanical microkeratome.^[12,19,20]^

This work focused not only on the histological changes that occurred in the extracted lenticules after Femto SMILE laser and free cap procedures but also on the alteration in the corneal proteins through DNA fragmentation analysis, comet assay, and FTIR spectroscopy.

The agarose gel electrophoresis offers a technique for the separation of the fragmented and the intact DNA fractions.^[27]^ In the free cap group, the appearance of a distinctive band in electrophoresis analysis without any fragmentation indicates the absence of apoptotic cells or DNA cleavage. In contrast, the Femto SMILE group pattern shows a change, appearing as an undefined band without any fragmentation. So far, the significance of this finding has not been clear as it does not enable us to determine the exact effect on the DNA of the lenticule. In this regard, the comet assay has been used to analyze DNA damage in the cells of the lenticules. The amount of DNA that migrates from the nucleus cavity measures the amount of DNA damage in the cell.^[28]^In this study, the comet image analysis compares the two signals obtained from undamaged and damaged DNA, the longer and brighter the tail, the higher the level of damage. Based on this result, the Femto SMILE laser group has more DNA breaks than the free cap group. The DNA breaks can be induced by many factors such as exposure to ultraviolet, ionizing radiation, X-rays, chemicals, pollutants, and oxidative stress.^[29,30,31,32,33]^ High oxidative stress is the most probable cause for the elevated level of DNA breaks. It ensues under a condition of light exposure, high energy consumption, or a weakening of coping ability.^[34,35]^These factors result in abnormality in the cellular metabolic process.^[36]^ Furthermore, this finding suggested that the Femto SMILE group was exposed to the laser photodisruption effect. In this process, the evaporation of microscopic volumes of tissue results in cavitation gas bubbles (carbon dioxide and water).

The vibrational frequency obtained from the FTIR spectra for the lenticules is a measure of the changes in the molecular structure. Hence, lenticular proteins were analyzed for the different spectral regions, namely NH–OH stretching, CH stretching, fingerprint, and amide I regions. Consequently, the FTIR analysis results for the two groups differ from each other. Nevertheless, the data obtained from FTIR spectroscopy are broadly consistent with the agarose gel electrophoresis and the comet assay. The significant difference between the NH–OH wavenumber in the free cap and Femto SMILE groups may be related to the instability in the formation of hydrogen bonds. On the other hand, the Femto SMILE group showed significant change in the wavenumber of the CH stretching region which appeared at 2962 cm ${}^{-1}$ in the free cap group and at 2938 cm ${}^{-1}$ in the Femto SMILE group. The decline in the wavenumber of the Femto SMILE group indicates an environmental change leading to disorder in the lipid hydrocarbon chains. Moreover, the amide II band at 1558 cm ${}^{-1}$ was shifted to 1530 cm ${}^{-1}$ causing alteration in the ratios of amide II to amide I and reveals disorder in the proteins' secondary and tertiary structure which may cause unfolding, aggregation and proteins distortion.^[34]^ Furthermore, the change in ${}_{sym}$ PO ${}_{2}$ wavenumber from 1070 cm ${}^{-1}$ to 1056 cm ${}^{-1}$ may be related to spatial changes in the positions of the phosphate groups in the proteins helix. This change may lead to several changes in the nucleic acid content of phospholipids. Therefore, it probably reveals that initial changes in the secondary and tertiary structure of the proteins also may cause changes in genetic materials.^[37]^ Our findings may indicate that the Femto SMILE laser group has more significant DNA damage and fragmentation than the free cap group. Moreover, the slight alteration in the histological analysis and DNA analysis of the free cap group may also be related to many factors such as tissue processing, donor age, time of storage, and samples transportation.

These results are parallel with the results obtained with histological analysis and FTIR spectroscopy. Thus, the influence of the Femto SMILE laser may be due to the deep dispersion of the Femto laser into the corneal lamella. The closer the Femto laser is to the targeted area, the more exposure to the heat energy and the shock waves causes collateral damage to the cell layer.^[38]^ Thus, it may be considered as a risk factor that may affect the rest of the corneal lamella.

Although SMILE induced slight effects on corneal lamellar cells, the advantages of the Femto SMILE laser include the corneal biomechanical preservation, which results in excellent maintenance of the structure and low postoperative dry eye syndrome.^[10,39,40]^

Further studies on the impact of Femto SMILE laser surgery on corneal morphology and proteins using larger sample sizes, different lenticular depths, and laser parameters are essential.

In summary, the results of our research suggested that the Femto SMILE procedure induced more structural changes than those induced by a microkeratome cutting. In addition, Femto SMILE patients had a higher rate of DNA damage than the patients in the free cap group. The effect of the Femto SMILE laser technique on the DNA revealed by the corneal histological examination may be attributed to the deep penetration of high energy levels to the corneal layer. Future studies are obligatory on experimenting with using different frequencies, optimum energy, spot size, and spot distance to reach the best outcome from the Femto SMILE procedure. Moreover, additional investigations on the efficacy of administrating systemic antioxidants and DNA repair mechanism may protect the cornea from oxidative stress in the Femto SMILE patients.

##  Financial Support and Sponsorship

None.

##  Conflicts of Interest

None.
